# Clinical expertise shifts psychological distancing from effortful regulation to reduced engagement on internally oriented processing during multitasking

**DOI:** 10.3389/fnbeh.2026.1811561

**Published:** 2026-07-29

**Authors:** Kelssy Hitomi dos Santos Kawata, Shusaku Uruta, Airi Watanabe, Koki Ono, Shoichiro Amari, Hironori Nakatani, Kyosuke Nozawa, Mihoko Sasaka, Kimikazu Kashiwagi, Hiroshi Oyama

**Affiliations:** 1 Department of Clinical Information Engineering, Graduate School of Medicine, The University of Tokyo, Tokyo, Japan; 2 Graduate School of Interdisciplinary Information Studies, The University of Tokyo, Tokyo, Japan; 3 Faculty of Information Sciences and Graduate School of Information Sciences, Hiroshima City University, Hiroshima, Japan; 4 Department of Preventive Medicine and Public Health, School of Medicine, Keio University, Tokyo, Japan; 5 Department of Information Media Technology, School of Information and Telecommunication Engineering, Tokai University, Tokyo, Japan; 6 Division of Health Sciences, Graduate School of Medicine, The University of Osaka, Osaka, Japan; 7 Graduate School of Nursing, Kawasaki City College of Nursing, Kawasaki, Kanagawa, Japan; 8 Faculty of Nursing, Tokyo Ariake University of Medical and Health Sciences, Tokyo, Japan; 9 National College of Nursing, Tokyo, Japan

**Keywords:** coping, emotion regulation, expertise, fMRI, immersive virtual reality, multitasking, novice, nurses

## Abstract

**Background:**

Clinical decision-making often occurs under conditions of high cognitive load, emotional stress, and time pressure. Psychological distancing, an emotion-focused coping strategy involving the mental separation of oneself from emotionally stressful situations to reduce affective impact, supports affect regulation in such contexts; however, its neural implementation during realistic multitasking and the extent to which it is shaped by clinical expertise remain poorly understood.

**Methods:**

We investigated the neural correlates of psychological distancing during high-risk multitasking clinical scenarios in experienced nurses and nursing students. Participants first completed immersive virtual reality simulations of realistic clinical situations, followed by an auditory functional magnetic resonance imaging task derived from the same scenarios. Psychological distancing was assessed using a validated self-report measure, and brain–behavior associations were examined separately for experienced nurses and nursing students.

**Results:**

Although self-reported psychological distancing did not differ between groups, clear expertise-related neural differences were observed. In experienced nurses, higher distancing was associated with reduced activation in regions of the default mode network, including the angular gyrus, middle temporal gyrus, and precuneus, which are implicated in internally oriented cognitive and emotional processing. In contrast, nursing students exhibited increased activation restricted to the cerebellum, suggesting greater dependence on effortful and compensatory neural mechanisms during emotion regulation under multitasking demands.

**Conclusion:**

These findings provide evidence that clinical expertise may influence the neural implementation of psychological distancing, shifting it from effortful regulation toward reduced engagement of internally oriented processing, which may reflect a more automatized regulatory implementation. This neural process may support adaptive emotion regulation during decision-making under stress, highlighting a potential neurobehavioral mechanism through which expertise shapes performance in complex clinical environments.

## 1 Introduction

Clinical decision-making in healthcare routinely occurs under conditions of high cognitive load, emotional stress, and time pressure (Croskerry, 2009; Kahneman and Klein, 2009). Clinicians are required to manage multiple concurrent tasks, often amid frequent interruptions, while regulating emotional responses to patient suffering, uncertainty, and risk (Arora et al., 2010; Shanafelt et al., 2015). Failures in emotion regulation under such conditions can compromise decision quality, patient safety, and clinician well-being (Heyhoe et al., 2016; Jackson-Koku and Grime, 2019; Batanda, 2024). Accordingly, understanding how healthcare professionals cope with emotional demands during complex clinical situations is a central concern in cognitive neuroscience and clinical education, particularly given the need to sustain effective decision-making under stress.

Emotion-focused coping strategies have received increasing attention as mechanisms that allow individuals to manage emotional responses without necessarily altering external stressors (Lazarus, 1984; Gross, 1998). Among these strategies, psychological distancing has been identified as a key mechanism for regulating emotional responses to stress. Distancing involves cognitive operations that attenuate emotional reactivity by creating mental separation from emotionally salient events, commonly through adopting a detached or observer-like perspective (Ochsner and Gross, 2005; Buhle et al., 2014; Kohn et al., 2014). Experimental work has shown that distancing effectively reduces negative affect and physiological responses; however, when implemented deliberately, it often relies on effortful cognitive control processes, particularly in individuals with less efficient or less automatized regulatory strategies (Buhle et al., 2014; Kohn et al., 2014; Powers and LaBar, 2019).

Neuroimaging research has consistently linked psychological distancing and related emotion-regulation strategies to activity within the default mode network (DMN), including the angular gyrus (AnG), middle temporal gyrus (MTG), and precuneus (PCu) (Buckner et al., 2008; Spreng et al., 2009; Seghier, 2013; Buhle et al., 2014; Buckner and DiNicola, 2019). These regions support internally oriented cognitive processes such as self-referential thought, perspective taking, mental simulation, semantic interpretation, and the integration of internally generated representations (Spreng et al., 2009; Seghier, 2013; Buckner and DiNicola, 2019). During effortful emotion regulation, increased engagement of DMN regions is commonly observed, reflecting deliberate reinterpretation, reflective processing, and perspective shifting in response to emotional stimuli (Ochsner and Gross, 2005; Buhle et al., 2014; Morawetz et al., 2017). However, it remains unclear whether and how the neural implementation of distancing, particularly the recruitment of DMN-mediated internally oriented processes, varies as a function of clinical expertise.

Theoretical models of expertise propose that repeated training and real-world exposure lead to the proceduralization and automatization of cognitive and regulatory strategies, thereby reducing reliance on deliberative, reflective, and self-referential processing during performance under pressure (Ericsson and Lehmann, 1996; Chein and Schneider, 2005). Consistent with this view, large-scale network accounts of skilled performance suggest reduced engagement of DMN regions during skilled task execution, reflecting efficient suppression of internally oriented processes that may be unnecessary or even disruptive to goal-directed action (Buckner et al., 2008; Seghier, 2013). In clinical contexts, these models suggest that experienced practitioners may regulate emotion more implicitly, minimizing semantic-emotional elaboration and self-focused imagery while prioritizing externally relevant task demands.

Despite increasing interest in the neural basis of expertise, no studies to date have directly examined how psychological distancing is implemented at the neural level during realistic, high-risk clinical multitasking, nor how this neural implementation differs between experienced clinicians and novices. Moreover, behavioral self-report measures of coping may not fully capture expertise-related differences in the underlying neural mechanisms, as individuals with different levels of experience may endorse similar coping tendencies while deploying qualitatively distinct regulatory processes (Nisbett and Wilson, 1977; Ericsson and Lehmann, 1996; Gross and John, 2003; Buhle et al., 2014).

Investigating coping and decision-making in realistic clinical contexts presents substantial methodological challenges. Traditional laboratory paradigms often lack ecological validity, whereas real-world clinical environments are difficult to standardize and incompatible with neuroimaging constraints. Immersive virtual reality (IVR) provides a controlled method for simulating complex and emotionally salient clinical scenarios. In the present design, IVR was used to induce a standardized experiential context prior to neuroimaging. Neural responses were subsequently assessed using an auditory functional magnetic resonance imaging (fMRI) task derived from the same scenarios, allowing examination of cognitive–emotional processing associated with these experiences under experimentally constrained conditions. While this approach increases contextual relevance compared to abstract laboratory tasks (Lem et al., 2024; dos Santos Kawata et al., 2025), it does not constitute direct measurement of neural activity during immersive multitasking itself.

To address these gaps, the present study investigated how psychological distancing as an emotion-focused coping strategy relates to neural responses during multitasking clinical risk, and how this relationship is shaped by clinical expertise. Combining IVR simulations of realistic clinical scenarios with an auditory fMRI task based on identical scenarios, we compared experienced nurses (EN) and nursing students (NS). This approach allowed us to examine whether comparable self-reported distancing tendencies are supported by different neural mechanisms as a function of expertise.

Based on emotion-regulation and expertise frameworks, we hypothesized that, despite potentially comparable self-reported tendencies to use psychological distancing, EN would exhibit reduced engagement of DMN regions involved in internally oriented cognitive and emotional processing, reflecting a potentially more automatized implementation of emotion regulation. In contrast, psychological distancing in NS was expected to rely on more effortful and compensatory neural mechanisms.

## 2 Methods

### 2.1 Ethics statement

All participants provided written informed consent prior to participation and received monetary compensation. The study was approved by the University of Tokyo Research Ethics Committee (2019107NI) and conducted in accordance with the Declaration of Helsinki. The study protocol was preregistered at the University Hospital Medical Information Network Clinical Trials Registry (UMIN-CTR; UMIN000052131; registration date: 06/09/2023).

### 2.2 Participants

Forty-three participants were recruited from the Tokyo area between September and December 2023, including 21 experienced nurses [EN; 19 females; age (mean (SD)) = 38.38 (4.76) years] and 22 nursing students [NS; 19 females; age (mean (SD)) = 21.41 (0.50) years]. Inclusion criteria required participants to be native Japanese speakers and right-handed, with no neurological or psychiatric disorders, Magnetic Resonance Imaging (MRI) contraindications, claustrophobia, or pregnancy. NS were fourth-year students, and experienced nurses were required to have ≥5 years of clinical experience.

All 43 participants completed the demographic assessment, the coping-related multitasking IVR simulation, and the coping-related multitasking auditory fMRI task. However, because the IVR simulation automatically terminated when participants made an incorrect decision, only 33 participants 19 EN (17 female; age range = 30–50 years; [mean (SD)] = 38.21 (4.80) years) and 14 NS [13 female; age range = 21–22 years, (mean (SD)) = 21.36 (0.50) years] were able to complete the entire IVR scenario and thus provided valid decision-making responses. These 43 and 33 participants were included in the final behavioral and neuroimaging analyses (Table 1).

**Table 1:** Demographic characteristics of participants in the initial and fMRI samples [mean (SD)].

	All	EN	NS	*t*/X^2^	*p*-value
Initial sample	43	21	22		
Age (years)	29.70 (9.20)	38.38 (4.76)	21.41 (0.50)	−16.26	**
Sex (female/male)	38/5	19/2	19/3	0.18	0.67
JART score	29.70 (6.87)	30.81 (7.46)	28.64 (6.25)	−1.04	0.31
fMRI sample	33	19	14		
Age (years)	31.06 (9.20)	38.21 (4.80)	21.36 (0.50)	−15.19	**
Sex (female / male)	30/3	17/2	13/1	0.11	0.74
JART score	28.70 (7.13)	30.11 (7.28)	26.79 (6.72)	−1.34	0.19

SD, standard deviation; EN, experienced nurses; NS, nursing students; JART, Japanese Adult Reading Test, **p < 0.01.

### 2.3 Experimental overview

Participants first completed demographic and cognitive assessments, including the Japanese Adult Reading Test (JART). They then underwent a baseline fMRI session, followed by the coping-related multitasking IVR simulation (i.e., IVR simulation), post fMRI session, and finally distancing subscale of the Stress Coping Inventory (SCI). We designed two experimental tasks. One was a coping-related multitasking IVR simulation developed to capture behavioral and experiential responses during a dynamic clinical scenario, and the other was a distancing-as-a-coping multitasking auditory fMRI task designed to examine neural responses associated with distancing coping-related cognitive processing. Detailed descriptions of both tasks are provided in the following sections.

During the baseline and post-fMRI sessions, participants lay supine with foam pads and a headband to reduce motion. All instructions and the coping-related multitasking auditory fMRI task (i.e., auditory fMRI task) were delivered through MRI-compatible headphones. To preserve continuity with the IVR experience while remaining compatible with MRI constraints, participants wore a sleeping mask throughout scanning following removal of the Head Mounted Display (HMD). Introducing visual stimuli during fMRI could have altered the sensory context relative to the original IVR experience. Therefore, the task was presented auditorily to minimize additional visual influences and facilitate mental reconstruction of the previously experienced clinical scenarios.

Following completion of the baseline fMRI session, participants exited the MRI scanner and rested for approximately 5 min. Subsequently, they performed the coping-related multitasking IVR simulation while seated in a 360-degree rotating chair. The experimenter explained how to operate the HMD and handheld controller. Participants were instructed to discontinue the simulation if they experienced discomfort or cybersickness. Immediately after the IVR simulation, participants were instructed to close their eyes before the HMD was removed, after which they were blindfolded.

Participants were subsequently escorted back to the MRI scanner for the post fMRI session. The scanning setup and procedures were identical to those used in the baseline fMRI session. In both sessions, participants wore a sleeping mask and completed the distancing-as-a-coping multitasking auditory fMRI task. During the task, participants were instructed to imagine themselves in the described clinical scenarios and respond verbally to the two risk-related decisions. Following completion of the post auditory fMRI task, a high-resolution structural brain image was acquired.

Importantly, the IVR simulation and the fMRI task differed in modality and interaction demands. The IVR component involved real-time visuomotor engagement within an interactive environment, whereas neural measurements were obtained during a subsequent auditory task requiring imagination of the same scenarios without visual or motor input. Thus, the fMRI data do not capture neural activity during the immersive IVR experience itself, but rather reflect neural processes associated with recalling, reconstructing, and cognitively simulating those scenarios under controlled conditions.

### 2.4 Coping-related multitasking IVR simulation

#### 2.4.1 Apparatus

**Figure 1:**
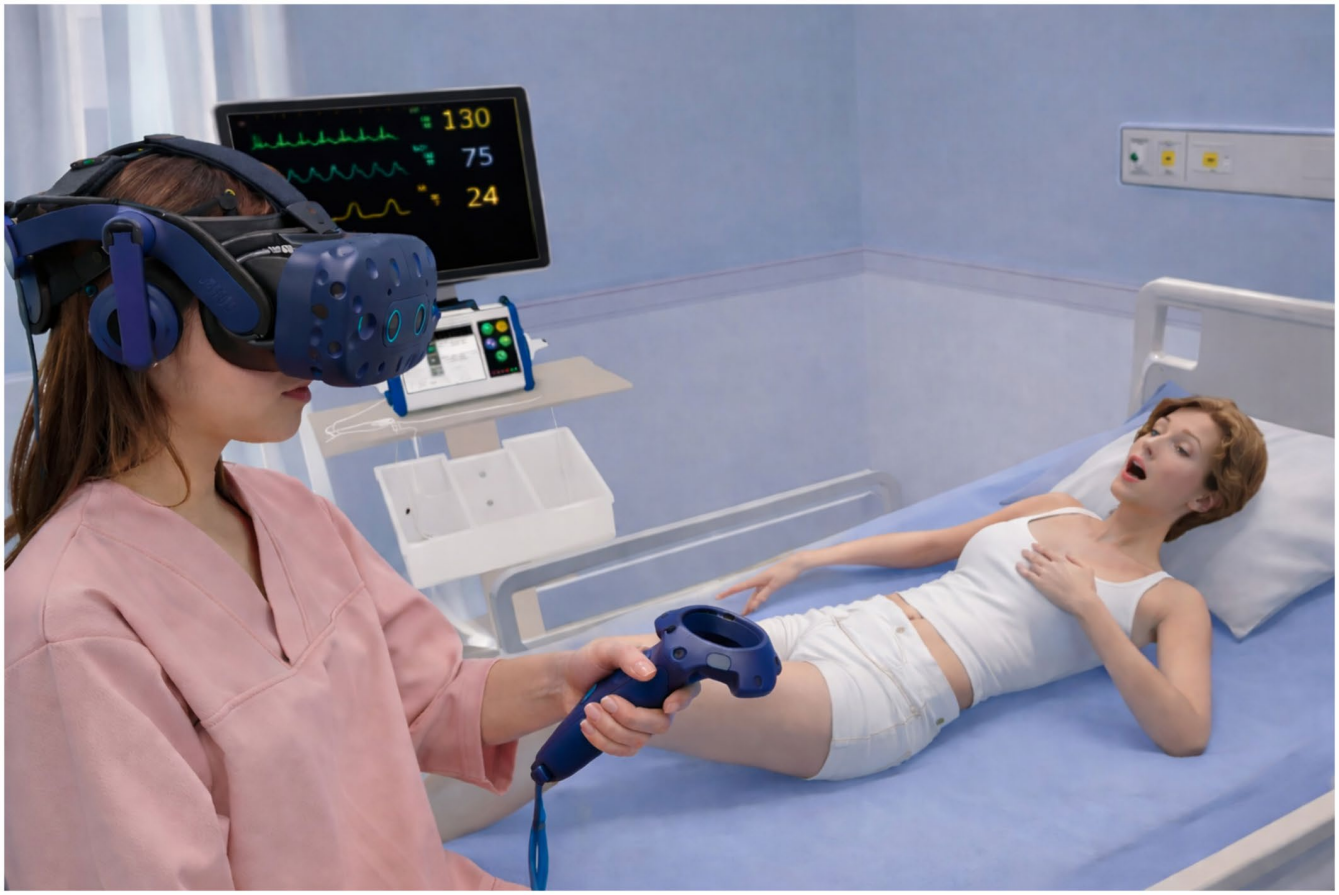
A depiction of the setup used for the coping-related multitasking IVR simulation and a screenshot of the virtual environment was developed using Unity version 2019.4.21f1 (https://unity.com/releases/editor/whats-new/2019.4.21) and the Blender software version 2.80 (https://www.blender.org/download/releases/). Virtual characters were obtained through the Unity Asset Store, a hub of freely available 3D anime-based models (https://assetstore.unity.com/). IVR, immersive virtual reality.

The IVR simulation was delivered using a High-Tech Computer Corporation (HTC) Vive Pro Eye HMD (90 Hz refresh rate; 1,440 × 1,600 resolution per eye; 110° field of view) with integrated headphones and an HTC Vive controller in the right hand (Figure 1). The IVR environment was developed in Unity (version 2019.4.21f1) (Unity 2019.4.21f1, 2019) and the Blender (version 2.80) (Blender, 2019) using C#. Virtual characters were obtained from the Unity Asset Store, which provides freely available 3D anime-style models (Asset Store, n.d.).

#### 2.4.2 IVR application

The IVR contents were adapted from a previously developed educational IVR system (Sato et al., 2020). Participants assumed the role of a nurse in a first-person virtual hospital and completed two sequential clinical scenarios requiring coping with the situation and making clinical risk decision.

The IVR simulation consisted of a single continuous run. Scene 1 and Scene 2 were each presented once in a fixed order, reflecting the original design of the educational system. After the instructional section, Scene 1 placed participants in a hospital room with a postoperative patient. During temperature assessment, a nurse call alerts them to another patient with an ostomy pouch leakage. At this point, the participant must deal with the situation by making the first clinical decision (“Decision 1”): “Choice 1”—continue the temperature assessment for the postoperative patient (correct), or “Choice 2”—prioritize the patient with the ostomy pouch leakage (incorrect). Selecting the incorrect choice terminated the simulation (Sato et al., 2020).

In Scene 2, a separate patient requests assistance changing clothes. The patient appears sweaty and distressed. Participant made a second clinical decision (“Decision 2”), choosing among: “Choice 1”— return to the patient with the ostomy pouch leakage (incorrect), “Choice 2”—call another nurse for assistance (acceptable), or “Choice 3”—further assess the sweating patient (acceptable) (Sato et al., 2020).

### 2.5 Assessments

#### 2.5.1 Demographic characteristics

Demographic information collected at baseline included age, sex, and JART (Matsuoka, 2002). The JART is a 50-item kanji reading test with established validity and reliability and is widely used to estimate Intelligence Quotient (IQ) (Matsuoka, 2002; Shimada et al., 2022). Higher scores indicate higher estimated IQ.

#### 2.5.2 Distancing measure of the Stress Coping Inventory (SCI)

The SCI distancing subscale (Japanese Institute of Health Psychology, 1996) assessed participants’ tendency to regulate stress through psychological distance. Participants first described a recent stressful event, then rated eight items (0–2 scale). The total score represented the distancing tendency.

The distancing subscale reflects a cognitive strategy characterized by psychological detachment, reframing, and reduced emotional involvement that is, creating distance between oneself and the event. Higher scores indicate greater use of this strategy. The SCI has demonstrated content validity in Japanese samples, including nurses (Taniguchi and Mizuno, 2016).

### 2.6 Distancing-as-a-coping multitasking auditory fMRI task

All participants considered for the final analyses completed an auditory fMRI task designed to simulate the same clinical scenarios presented in the IVR environment. A preliminary study with 16 nurses [12 female; clinical experience (mean (SD)) = 8.72 (9.48) years] determined the appropriate decision-making durations within the IVR scenario. Mean response times (5 s for Decision 1; 9 s for Decision 2) were incorporated into the auditory fMRI task timing structure.

During scanning, instructions and decision prompts were delivered through MRI-compatible headphones (SS3300; Avotec, Inc., Stuart, FL) without visual stimuli. Each block contained two trials (41 s for Decision 1; 40 s for Decision 2). In the Decision 1 trial, Choices 1 and 2 were presented for 14 s and 17 s, respectively, followed by a 5-s thinking period and a 5-s response interval. The Decision 2 trial began immediately afterward, with Choices 1, 2, and 3 presented for 14 s, 6 s, and 6 s, followed by 5 s to think and a 9-s verbal response period. Four such blocks were presented per session (i.e., baseline and post sessions) and were separated by rest periods (9 s), a 6-s general instruction cue, a 22-s contextual description, and a 4-s preparation period. Participants were instructed to listen carefully, imagine themselves in the described scenario, and provide verbal responses. Each session consisted of eight total trials and lasted approximately 10 min. The auditory fMRI task stimuli were delivered using PsychoPy v2021.2.3 (Peirce, 2007, 2009).

### 2.7 MRI data acquisition

All MRI data were collected using a 3-T MAGNETOM Prisma scanner (Siemens, Erlangen, Germany) with a 64-channel head coil. During the auditory fMRI task, we acquired 39 continuous slices of functional images per volume (field of view (FOV) = 192 × 192 mm^2^; matrix = 64 × 64; slice thickness = 3 mm; slice gap = 0.75 mm) with an echo planar imaging (EPI) pulse sequence (repetition time (TR) = 2000 ms; echo time (TE) = 25 ms; flip angle (FA) = 90°). A total of 628 volumes were obtained for each of the baseline and post sessions. Slices were tilted 30° from the anterior commissure–posterior commissure line to reduce susceptibility artifacts.

High-resolution structural images were acquired using a T1-weighted magnetization-prepared rapid acquisition gradient echo (MPRAGE) sequence (voxel size = 1 × 1 × 1 mm^3^; TR = 2000 ms; TE = 2.9 ms; FA = 9°; FOV = 256 × 256 × 256 mm^3^). All scans were visually inspected for image quality and motion. Participants were reminded to remain relaxed and avoid falling asleep during scanning.

### 2.8 Analysis

#### 2.8.1 Behavioral data analysis

Normality of SCI distancing scores within each group (EN and NS) was assessed using the Shapiro–Wilk test. Because the data were normally distributed, group differences in continuous variables were examined using independent samples t-tests, and categorical variables were compared using chi-square tests. Analyses were conducted using Python version 3.12.9 with statistical significance set at *p* < 0.05.

#### 2.8.2 fMRI data analysis

Preprocessing was performed using statistical parametric mapping software (SPM12; Wellcome Department of Imaging Neuroscience; Institute of Neurology, London, United Kingdom) implemented in MATLAB R2017b (MathWorks Inc., Natick, MA, USA). Preprocessing included correction for head motion, co-registration to the anatomical image, spatial normalization using the anatomical image and the Montreal Neurological Institute (MNI) template, and smoothing using a Gaussian kernel with full width at half-maximum of 6 mm. None of the registrations of the participants’ images differed between the baseline and post fMRI sessions.

A conventional two-level fMRI analysis was conducted using SPM12. As a first-level analysis, task-related activation and deactivation were estimated for each participant using a general linear model (GLM). For each participant, baseline and post fMRI sessions were modeled within the GLM framework. The design matrix included task regressors representing the auditory fMRI task periods. These regressors were constructed by modeling the onset and duration of each trial and were convolved with the canonical hemodynamic response function (HRF) provided by SPM12. Trials were modeled as blocks, corresponding to the presentation of the auditory clinical scenarios. The same regressor structure was applied to both baseline and post sessions to enable direct within-subject comparisons. First-level contrast images were computed to estimate condition-specific effects, including post < baseline, post > baseline simulation contrasts. Six estimated head motion parameters were included in the GLMs as confounding factors.

In the second-level analysis, the neural correlates of individual differences in distancing scores were estimated using a voxel-by-voxel multiple regression analysis of the coping-related multitasking auditory fMRI task simulation for EN and NS groups separately. For each negative and positive auditory fMRI task simulation response, the total distancing scores for the EN and NS simulations were included as independent variables of interest. In the contrasts, age, sex, and JART scores were included as covariates.

Primary whole-brain analyses used a voxel-wise cluster-forming threshold of *p* < 0.001 (uncorrected) with cluster-level family-wise error (FWE) correction at *p* < 0.05. To examine cross-group correspondence, the peak voxels identified in each group were tested in the other group using a region-of-interest (ROI) approach. These follow-up ROI analyses were conducted using a liberal uncorrected threshold of *p* < 0.01 and were considered exploratory. Anatomical labels were assigned using the SPM Anatomy Toolbox (Eickhoff et al., 2005).

## 3 Results

### 3.1 Demographic characteristics

Demographic characteristics are presented in Table 1. Independent samples t-tests indicated significant age differences between EN and NS in both the initial and fMRI samples [Initial sample: EN (mean (SD)) = 38.38 (4.76) years; NS (mean (SD)) = 21.41 (0.50) years; *t* = −16.26, *p* < 0.01; fMRI sample: EN (mean (SD)) = 38.21 (4.80) years; NS (mean (SD)) = 21.36 (0.50) years; *t* = −15.19, *p* < 0.01]. In contrast, no significant group differences were observed for sex or JART scores in either sample (*p* > 0.05).

### 3.2 Effect of the distancing measure of the SCI between EN and NS

To examine whether clinical experience influences participants’ tendency to regulate stress through psychological distancing, independent-samples t-tests were conducted to compare the mean total distancing scores between EN and NS (Table 2). No significant group differences were observed in either sample [Initial sample: EN (mean (SD)) = 5.90 (2.68); NS (mean (SD)) = 6.95 (2.95); *p* = 0.23; fMRI sample: EN (mean (SD)) = 5.68 (2.54); NS (mean (SD)) = 6.64 (3.30); *p* = 0.35]. Effect size estimates indicated small differences between groups in both samples [initial sample: Cohen’s d = −0.37, 95% CI (−0.97, 0.23); fMRI sample: Cohen’s d = −0.33, 95% CI (−1.03, 0.37)]. These results suggest a consistent but small effect, with higher distancing scores in NS compared to EN. However, the wide confidence intervals, which include zero, indicate substantial uncertainty regarding the true effect size. Accordingly, the results are compatible with a range of effects from negligible to small-to-moderate in magnitude, and the absence of statistical significance may reflect limited statistical power rather than the absence of a true effect.

**Table 2:** Independent samples *t*-test results comparing differences between experienced nurses and nursing students in the distancing total score of the Stress Coping Inventory (SCI) [mean (*SD*)].

	EN	NS	*t*	*p*-value
Distancing total score				
Initial sample (*n* = 43)	5.90 (2.68)	6.95 (2.95)	1.22	0.23
fMRI sample (*n* = 33)	5.68 (2.54)	6.64 (3.30)	0.94	0.35

SD, standard deviation; EN, experienced nurses; NS, nursing students; IVR, immersive virtual reality.

### 3.3 Specific neural activation in EN during the distancing-as-a-coping multitasking auditory fMRI task

**Figure 2:**
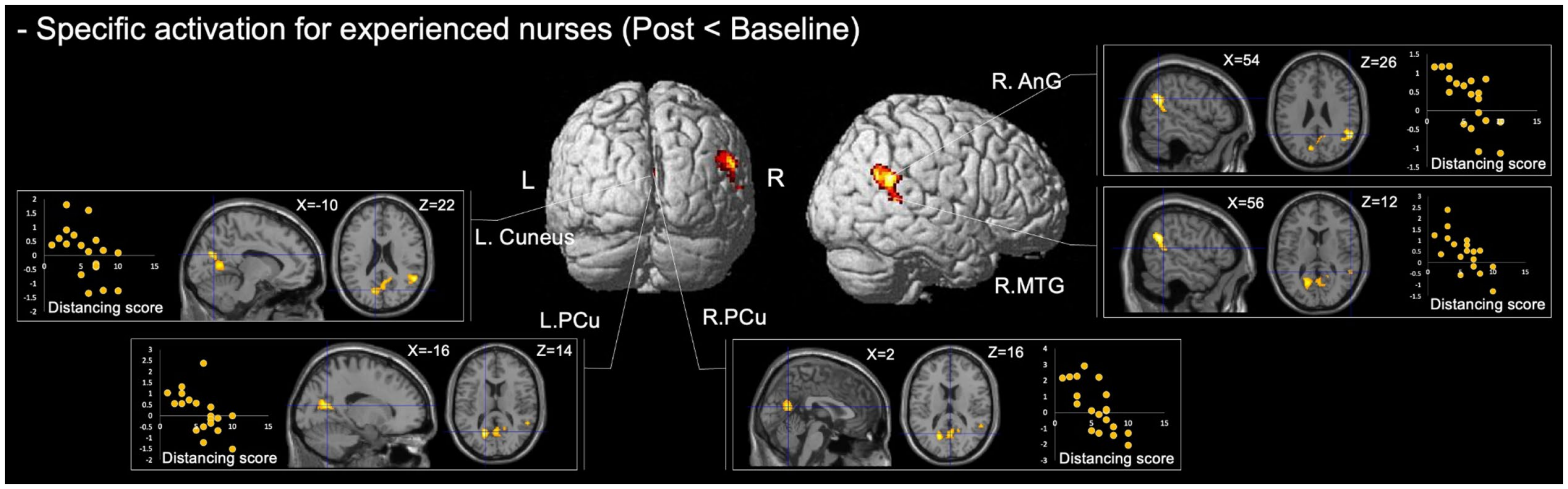
The distance-related activation before the coping-related multitasking IVR simulation is specific to experienced nurses. The voxel-wise statistics used an uncorrected *p* < 0.001 for the cluster-forming threshold, set at a family-wise error-corrected *p* < 0.05 for cluster extent. Other details are the same as for Table 3. L, left; R, right; PCu, precuneus; AnG, angular gyrus; MTG, middle temporal gyrus.

Significant negative activation (post < baseline) was observed in EN in the R. AnG, R. MTG, bilateral PCu, and L. cuneus during the coping-related multitasking auditory fMRI task. Voxel-wise statistics were thresholded at uncorrected *p* < 0.001 for cluster formation, with a family-wise error (FWE)–corrected cluster extent threshold of *p* < 0.05 (Table 3; Figure 2). No significant positive activation (post > baseline) was identified in the EN group.

**Table 3:** The significant effect of coping-related multitasking IVR simulation for experienced nurses and nursing students in distancing score (*n* = 33).

Brain areas	Peak			Cluster		
	x	y	z	*t*	k	*p*
Post < baseline for EN						
R. AnG	54	−52	26	6.50	340	< 0.001
R. MTG	56	−46	12	4.59		
L. PCu	−16	−62	14	5.64	699	< 0.001
L. Cuneus	−10	−70	22	5.04		
R. PCu	2	−62	16	4.85		
Post > baseline for EN						
*n.s.*						
Post < baseline for NS						
*n.s.*						
Post > baseline for NS						
R. Cerebellum Exterior	26	−80	−28	8.14	261	<0.001

The coordinates (x, y, z) and *t*-value of the peak activation, as well as the size and *p*-value of the clusters, are given for the post < baseline and post > baseline of distancing score during the coping-related multitasking auditory fMRI task simulation in EN and NS. fMRI, functional magnetic resonance imaging; EN, experienced nurses; R, right; AnG, angular gyrus; MTG, middle temporal gyrus; L, left; PCu, precuneus; n.s., not significant; NS, nursing students.

### 3.4 Specific neural activation in NS during the distancing-as-a-coping multitasking auditory fMRI task

**Figure 3:**
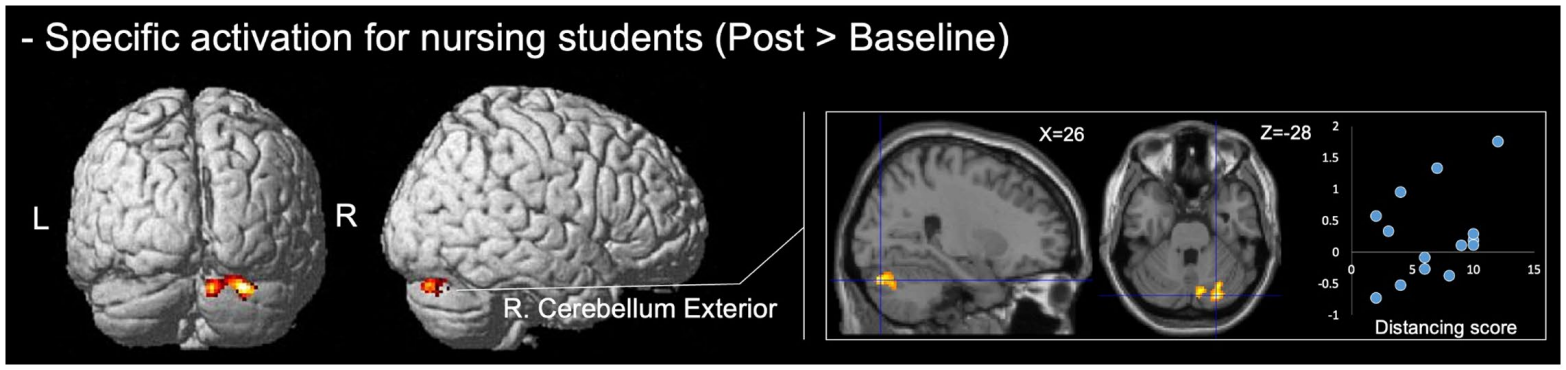
The distance-related activation after the coping-related multitasking IVR simulation is specific to nursing students. The voxel-wise statistics used an uncorrected *p* < 0.001 for the cluster-forming threshold, set at a family-wise error-corrected *p* < 0.05 for cluster extent. Other details are the same as for Table 3. L, left; R, right.

The whole-brain contrast for NS (post > baseline) during the coping-related multitasking auditory fMRI task revealed significant positive activation in the R. cerebellum exterior. Voxel-wise statistics were thresholded at uncorrected *p* < 0.001 for cluster formation, with a family-wise error (FWE)–corrected cluster extent threshold of *p* < 0.05 (Table 3; Figure 3). No significant negative activation (post < baseline) was identified in the NS group. In addition, no common activation between EN and NS was detected even when applying a liberal ROI threshold of uncorrected *p* < 0.01.

## 4 Discussion

This study examined how psychological distancing, an emotion-focus coping strategy, relates to neural responses during multitasking clinical risk, and how this relationship is shaped by clinical expertise. To this end, we combined an immersive IVR exposure with a subsequent auditory fMRI task derived from the same scenarios, enabling investigation of how coping-related processes elicited in a realistic context are represented during later neural simulation. Importantly, these two types of exposures involve partially overlapping but distinct cognitive processes: whereas the IVR exposure entails real-time visuomotor interaction within a dynamic environment, the fMRI task relies on internally generated representations rather than real-time interaction. We compared EN and NS to clarify how expertise modulates neural engagement under cognitively and emotionally demanding conditions. At the behavioral level, no significant group differences were observed in self-reported distancing scores, suggesting that the general tendency to endorse distancing as a coping strategy does not vary systematically with clinical experience alone. However, despite comparable subjective self-reports, the two groups exhibited clearly distinct neural response patterns during the distancing-as-a-coping multitasking auditory fMRI task. This dissociation indicates that clinical expertise modulates how psychological distancing is implemented at the neural level, rather than whether it is endorsed behaviorally, highlighting a divergence between subjective coping tendencies and their underlying neural mechanisms.

### 4.1 Expertise-related neural deactivation during distancing-as-a-coping multitasking

The neural findings associated with distancing-as-a-coping multitasking suggest that EN implement psychological distancing with reduced engagement of internally oriented processes, a pattern that may be consistent with a more automatized, but does not by itself constitute direct evidence of neural efficiency. Prior work has emphasized that reduced activation can reflect either neural efficiency or task-related suppression/disengagement, depending on behavioral context and performance outcomes (Poldrack, 2000; Chein and Schneider, 2005; Kelly and Garavan, 2005). In EN, higher distancing scores were negatively associated with activity in the R. AnG, R. MTG, bilateral PCu, and L. cuneus regions previously implicated in emotion regulation (Cavanna and Trimble, 2006; Buhle et al., 2014; Kohn et al., 2014; Morawetz et al., 2017; Sokołowski et al., 2022). In addition, the R. AnG, R. MTG and bilateral PCu are core components of the DMN and are centrally involved in internally oriented processes such as perspective taking shifting, self-referential imagination, semantic interpretation and integration of internally generated representations (Spreng et al., 2009; Seghier, 2013; Buckner and DiNicola, 2019). Further, EN exhibited reduced activation in the L cuneus, a region implicated in visual–emotional processing (Sokołowski et al., 2022). Although the L. cuneus is not a core component of the DMN, its decreased activation in EN suggests attenuated visual–emotional reactivity, consistent with a more automatic implementation of emotion regulation during psychological distancing.

Reduced R. AnG activity suggests diminished engagement of internally oriented perspective taking shifting and semantic-emotional integrative processes in EN. The R. AnG is a key DMN hub supporting self–other distinction, perspective taking shifting, and the integration of internally generated representations (Spreng et al., 2009; Seghier, 2013; Utevsky et al., 2014; Buckner and DiNicola, 2019). Its deactivation in EN may indicate that experienced clinicians require less conscious semantic–emotional integration and reduced self–patient perspective shifting when regulating emotions during risk-related clinical situations. This interpretation aligns with expertise models proposing that repeated training and real-world exposure reduce reliance on deliberative and reflective processing, favoring more goal-directed strategies under pressure (Ericsson and Lehmann, 1996; Chein and Schneider, 2005). Consistent with this view, large-scale network accounts of skilled performance report reduced engagement of self-referential and reflective systems during skilled performance (Buckner et al., 2008; Seghier, 2013). Moreover, emotion-regulation research shows that psychological distancing recruits the AnG more strongly when implemented effortfully, particularly in individuals with less effective regulatory strategies (Ochsner and Gross, 2005; Buhle et al., 2014; Kohn et al., 2014; Denny et al., 2015). Together, these findings suggest that R. AnG deactivation in EN may reflect suppression of internally oriented perspective taking shifting and semantic-emotional integrative processes to prioritize externally relevant task demands and action selection.

Deactivation of the R. MTG suggests an attenuation of internally oriented interpretation of semantic–emotional processes during clinical decision-making. EN may suppress conceptual elaboration to maintain a task-focused, action-oriented processing mode. The MTG is centrally involved in semantic processing, narrative comprehension, social–emotional interpretation, and the integration of conceptual knowledge with contextual meaning, forming part of the DMN supporting internally oriented interpretation processes (Binder et al., 2009; Xu et al., 2015; Buckner and DiNicola, 2019). Further, the MTG involvement in social emotion recognition and interpersonal emotion regulation has also been well documented (Arioli et al., 2021; Wang et al., 2024). In EN, reduced R. MTG activity may reflect attenuation of semantic–emotional interpretation that could otherwise amplify affective load. This pattern is consistent with expertise models suggesting that experts rely less on explicit, knowledge-based reasoning and more on rapid pattern recognition and automatized responses under time pressure (Ericsson and Lehmann, 1996; Chein and Schneider, 2005). In the context of psychological distancing, R. MTG deactivation may reflect an attenuated suppression of semantic–emotional elaboration, enabling emotion regulation without engaging in effortful reinterpretation or meaning-making during complex clinical decision-making. Thus, R. MTG deactivation in EN may represent an adaptive neural mechanism of expertise, reflecting reduced suppression of semantic-emotional interpretation processing in favor of goal-directed decision-making during complex and high-risk clinical situations.

Similarly, reduced bilateral PCu activity suggests suppression of internally oriented self-referential imaginative process in EN. The PCu supports mental imagery, episodic memory retrieval, perspective taking, and internally generated simulations (Cavanna and Trimble, 2006; Buckner et al., 2008; Buckner and DiNicola, 2019). Deactivation of the bilateral PCu may indicate that EN down-regulate self-focused imaginative processes to maintain attentional resources on externally relevant clinical demands. This interpretation is consistent with emotion-regulation studies showing that effortful distancing recruits DMN regions, including the PCu, when explicit cognitive control over emotion is required (Ochsner and Gross, 2005; Buhle et al., 2014; Kohn et al., 2014; Morawetz et al., 2017). In contrast, expertise theories emphasize automatization through repeated exposure, resulting in diminished reliance on deliberative and introspective processing during decision-making under pressure (Ericsson and Lehmann, 1996; Chein and Schneider, 2005). Thus, bilateral PCu deactivation in EN likely reflects a neural pattern associated with expertise, reflecting diminished self-referential imaginative processing and more automatic regulation of emotional interference during multitasking clinical risk.

Finally, reduced activation of the L. cuneus in EN suggests attenuation of visual–emotional processing. Although the task was auditory, prior work indicates that the cuneus contributes to visual imagery and emotionally salient perceptual processing (Sokołowski et al., 2022). Therefore, L. cuneus reflect reduced engagement of emotionally driven visual imagery during imagined clinical scenarios, further supporting the interpretation of reduced engagement of emotion-related processes in experts.

Importantly, reduced activation in DMN regions should not be interpreted as a direct marker of neural efficiency. Such reductions may also reflect alternative processes, including task disengagement, differences in cognitive strategy, or diminished emotional or self-referential engagement during the task. Without direct behavioral neural coupling or performance-based indices, it is not possible to determine whether reduced DMN activity reflects more efficient processing or reduced task involvement. Accordingly, the present findings are more conservatively interpreted as reflecting differences in task-related neural engagement associated with psychological distancing, rather than definitive evidence of neural efficiency. Thus, rather than indexing neural efficiency per se, reduced DMN activity in the present study is more conservatively interpreted as reflecting decreased engagement of internally oriented processes during task performance.

### 4.2 Non-expertise-related neural activation during distancing-as-a-coping multitasking

In contrast to EN, NS exhibited activation exclusively in the R. cerebellar exterior, which may reflect increased cognitive effort when managing multitasking clinical decisions in the absence of extensive clinical experience. Although traditionally associated with motor control, the cerebellum has increasingly been recognized for its role in higher-order cognitive functions, including temporal prediction, error-based learning, and the coordination of complex cognitive operations under conditions of uncertainty (Ito, 2008; Buckner, 2013; Sokolov et al., 2017). Consistent with these perspectives, recent work has shown that the R. cerebellar exterior, more specifically the R. cerebellar Crus I and II, demonstrates significant increases in activity under high cognitive load, even during non-motor linguistic and executive tasks (Nakatani et al., 2023). This finding suggests that cerebellar recruitment may reflect compensatory processing under demanding conditions. The absence of widespread cortical activation or deactivation in NS, together with the lack of overlap with EN even under liberal statistical thresholds, suggests that NS employ qualitatively different neural strategies during distancing-as-a-coping multitasking. Rather than suppressing internally oriented cognitive processing, as observed in EN, NS may recruit auxiliary systems, such as the R. cerebellar exterior, to support performance during multitasking clinical decision-making. This activation is consistent with prior findings showing that novice often engage compensatory or effortful neural recruitment to meet task demands, reflecting less efficient processing under conditions of pressure or uncertainty (Ericsson and Lehmann, 1996; Poldrack, 2000; Chein and Schneider, 2005; Kelly and Garavan, 2005).

Importantly, these neural differences emerged despite the absence of group differences in self-reported distancing scores. This dissociation suggests that EN may deploy psychological distancing more implicitly or automatically, whereas NS rely on more effortful and compensatory mechanisms. Such findings align with models of expertise emphasizing proceduralization and automatization of regulatory strategies through repeated real-world exposure (Ericsson and Lehmann, 1996; Chein and Schneider, 2005), as well as with emotion-regulation frameworks distinguishing effortful from more automatic forms of emotion regulation (Ochsner and Gross, 2005).

Accordingly, the observed neural patterns above and in the previous section should be interpreted as reflecting the cognitive and emotional reconstruction of prior IVR experiences, rather than direct neural responses to immersive, real-time multitasking clinical risk.

### 4.3 Limitations and future directions

Several limitations should be considered when interpreting the present findings. First, due to MRI constraints on head and body movement and the incompatibility of standard Virtual Reality (VR) equipment, the IVR experience could not be performed inside the MRI scanner. Instead, neural activity was measured during an auditory fMRI simulation that relied on participants’ imagination of previously experienced IVR scenarios. Therefore, the observed neural responses likely reflect secondary representational processes rather than real-time immersive multitasking. Although mental imagery can recruit overlapping neural systems with perception (Dijkstra et al., 2017; Pearson, 2019), future studies using MRI-compatible VR systems would allow more direct investigation of brain activity during immersive clinical performance.

Second, the sample was restricted to participants who completed the full IVR scenario, resulting in a reduced sample size and potentially limiting generalizability. However, this ensured that neural responses reflected full engagement with the clinical decision-making sequence. Importantly, a *post hoc* power analysis (*α* = 0.05) based on the current sample size (EN = 19, NS = 14) indicated a statistical power of 0.28 when assuming a medium effect size (*d* = 0.50). This suggests that the present study was underpowered to detect effects of moderate magnitude. Accordingly, non-significant findings should be interpreted with caution, as they may reflect an increased likelihood of Type II error rather than the absence of true effects. The assumption of a medium effect size was based on conventional benchmarks (Cohen, 1988) and is consistent with prior research on prelicensure nursing students in simulated clinical tasks (Shinnick et al., 2011). In addition, the exclusion of participants who were unable to complete the IVR task introduces the possibility of selection bias. The final sample may be skewed toward individuals with greater tolerance or adaptability to immersive environments, potentially limiting generalizability to broader clinical populations. Future studies should aim to reduce these exclusions or better examine why some participants are unable to progress to the next phase of the IVR task, in order to better understand differences in engagement and performance.

Third, the study focused specifically on psychological distancing, and other relevant individual differences such as trait anxiety, empathy, or prior VR experience were not explicitly modeled. These factors may contribute to variability in neural responses and should be incorporated in future research. Longitudinal and training-based designs would also help clarify how the neural implementation of distancing develops with clinical experience.

Fourth, a key limitation of the present study is the lack of direct behavioral–neural linkage. Distancing was assessed using the Distancing subscale of the SCI, which captures a trait-like propensity to adopt psychological distancing strategies in everyday situations, rather than state-dependent regulatory processes during the experimental task. Accordingly, the observed neural activations should be interpreted as reflecting individual differences in habitual coping tendencies, rather than direct evidence of successful emotion regulation or decision performance within the scanner. Moreover, the absence of task-based behavioral indices such as decision accuracy, response latency, or trial-by-trial performance variability limits our ability to determine whether reduced activity in DMN regions is associated with improved regulatory efficiency or task performance. Future research should integrate behavioral performance measures collected during scanning and examine brain–behavior correlations to directly test whether distancing-related neural activations predict regulation success and decision outcomes under stress.

Finally, an important consideration is the substantial age difference between EN and NS groups, which may act as a confounding factor. Although age was statistically controlled, aging is associated with changes in DMN function, emotion regulation, and cognitive control (Mather et al., 2004; Ferdinand and Czernochowski, 2018; Baptista et al., 2026). Thus, reduced DMN engagement in EN may partly reflect age-related processes rather than expertise alone. Nonetheless, because age and expertise are inherently confounded in the present sample, their independent contributions cannot be fully separated. Future studies using age-matched controls, longitudinal designs, or training interventions are needed to disentangle developmental and expertise-related effects.

## 5 Conclusion

This study demonstrates that psychological distancing as an emotion-focused coping strategy during multitasking clinical risk is associated by distinct neural mechanisms as a function of clinical expertise. Although EN and NS may report comparable tendencies to use distancing, the neural correlates associated with distancing showed non-overlapping brain regions patterns across groups. In EN, higher distancing scores are associated with reduced activation in DMN regions implicated in internally oriented cognitive and emotional processing, including the R. AnG, R. MTG, and bilateral PCu. These deactivations suggest that, with increasing clinical expertise, psychological distancing is implemented through reduced engagement of internally oriented processes, which may reflect a more automatized regulatory pattern. Such neural modulation may facilitate the prioritization of externally relevant task demands during complex clinical situations.

In contrast, distancing in NS appears to rely on more effortful cognitive coordination, reflected by increased activation of the R. cerebellar exterior. Together, these findings suggest that clinical expertise transforms the neural implementation of psychological distancing from a cognitively and emotionally demanding process in NS to a more automatic regulatory pattern, which may reduce reliance on effortful processing in experienced practitioners. Notably, these results should be interpreted within the constraints of an indirect neuroimaging paradigm that captures simulated rather than real-time immersive processing. Demonstrating expertise-dependent differences in the neural substrates of coping-related distancing, this study provides new insight into the neural basis of adaptive multitasking under stress in healthcare settings and highlights the importance of efficient suppression of unnecessary internally oriented processing during critical clinical decision-making.

## Data Availability

The datasets presented in this article are not readily available because the datasets generated and analyzed during this study are subject to restrictions. The data contain sensitive human participant information and are protected under the conditions of approval granted by the institutional ethics committee. The approved protocol prohibits public data sharing, including the release of fully anonymized datasets, in order to safeguard participant confidentiality and privacy. Data may be made available to qualified researchers upon reasonable request and only after obtaining appropriate institutional ethical approval. Requests to access the datasets should be directed to KS, kawatakelssy@gmail.com.
